# Wedge-Filtering of Geomorphologic Terrestrial Laser Scan Data

**DOI:** 10.3390/s130202579

**Published:** 2013-02-20

**Authors:** Helmut Panholzer, Alexander Prokop

**Affiliations:** Department of Structural Engineering and Natural Hazards, Institute of Mountain Risk Engineering, BOKU-University of Natural Resources and Applied Life Sciences, Peter Jordan-Str. 82, 1180 Vienna, Austria; E-Mail: alexander.prokop@boku.ac.at

**Keywords:** terrestrial laser scanning, filtering, wedge

## Abstract

Terrestrial laser scanning is of increasing importance for surveying and hazard assessments. Digital terrain models are generated using the resultant data to analyze surface processes. In order to determine the terrain surface as precisely as possible, it is often necessary to filter out points that do not represent the terrain surface. Examples are vegetation, vehicles, and animals. Filtering in mountainous terrain is more difficult than in other topography types. Here, existing automatic filtering solutions are not acceptable, because they are usually designed for airborne scan data. The present article describes a method specifically suitable for filtering terrestrial laser scanning data. This method is based on the direct line of sight between the scanner and the measured point and the assumption that no other surface point can be located in the area above this connection line. This assumption is only true for terrestrial laser data, but not for airborne data. We present a comparison of the wedge filtering to a modified inverse distance filtering method (IDWMO) filtered point cloud data. Both methods use manually filtered surfaces as reference. The comparison shows that the mean error and root–mean-square-error (RSME) between the results and the manually filtered surface of the two methods are similar. A significantly higher number of points of the terrain surface could be preserved, however, using the wedge-filtering approach. Therefore, we suggest that wedge-filtering should be integrated as a further parameter into already existing filtering processes, but is not suited as a standalone solution so far.

## 1. Introduction

Laser scanning provides point-sample elevation data, which enables the automated and fast generation of Digital Elevation Models (DEM) that can provide information on the morphological features of terrain, vegetation and buildings. A generic DEM normally implies elevations of the terrain (bare earth z-values) void of vegetation and manmade features [1]. To obtain a correct DEM all non-ground points have to be filtered out. The problem of segregating canopy and ground laser returns in laser scanning data is widely known.

Different approaches for filtering laser scanning data exist: the auto-regressive process [2], mathematical morphology [3], method of least squares, robust interpolation [4], convex-concave cover [5], and procedures that use, based on a triangular meshing (TIN) of the DEM, the local terrain inclination as filter criterion [6,7], gridding methods in which a grid DEM is calculated trough including gradient based height values determined in a hierarchical data pyramid [8–10], method of multiscale curvature classification [11].

These methods filter data obtained from airborne laser scanning. Aerial surveys are usually carried out directly over the site and provide relatively equally distributed measuring data. In contrast, terrestrial laser scans yield very irregularly distributed measuring data. First, because the distance between measured points increases in proportion to the measuring distance; second, it is difficult to avoid shadows caused by obstacles, such as trees, shrubs, or buildings. It is therefore more difficult to filter ground and non-ground points to calculate a digital elevation model (DEM). Currently, the following steps are recommended by different authors [12]: (a) manual cleaning of the TLS datasets, *i.e.*, removal of non-ground points, for example vegetation, wires and mobile objects. This step is time consuming, but necessary in most cases; (b) sometimes automatic algorithms for filtering non-ground points may be applied, looking either for differences in geometry [13,14] or in intensity of the returned signal [15]. The algorithms usually succeed in filtering of trees, but often fail to filter small plants and bushes, which need to be removed manually; (c) instrumental errors, *i.e.*, scattering of the TLS measurements around their true value, should be corrected when accurate measurements are necessary [16], for example noise reduction by filtering or averaging [17,18]. Prokop and Panholzer [14] propose to combine the principle of the robust interpolation with the process of morphological opening (IDWMO). The wedge-filtering method we describe here has a different approach than the existing algorithms, but can only be used for terrestrial laser scanning data.

## 2. Methods

**Figure 1.**
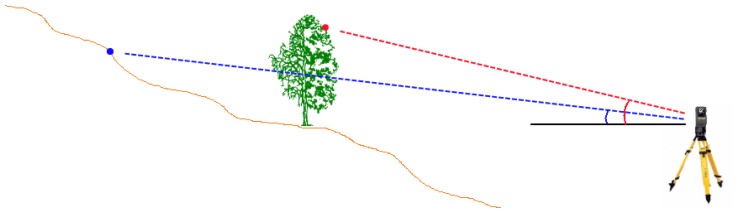
Cross-section of a terrestrial laser scan showing connections between laser scanner and recorded points.

In a static terrestrial laser scan, all measured points must be visible from the laser source of the scanner. Connecting each point with the laser source creates a line between the two, given that there were no obstacles along these lines. In case two connecting lines have the same horizontal angle, the connecting line with the larger vertical angle and smaller measuring distance (Figure 1, red line) compared to the connecting line with the smaller vertical angle and greater measuring distance (Figure 1, blue line) cannot lead to a ground point. Consequently, a point cannot be a ground point if there is a more distant point with a smaller vertical angle in the same direction.

**Figure 2.**
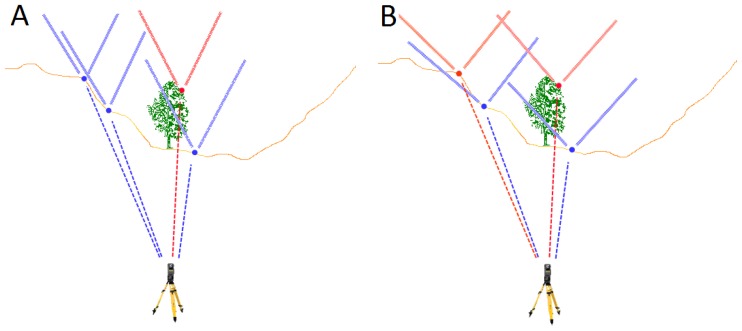
Frontal view of a terrestrial laser scan recording. Marked is the V-shaped elimination area. The blue dots are further away from the laser scanner than the red dots. Part **A** shows a larger filter angle than part **B**.

Only in a few cases, and often depending on the angular resolution of the scanner, two connecting lines have the same horizontal angle. To assess whether a point with a small vertical angle and a large measuring distance identifies another point with a greater vertical angle and a smaller measuring distance as a non-ground point, an elimination area must be defined in regard to the deviation of the horizontal angle. If the horizontal deviation increases proportionally to the difference of the vertical angle, the elimination area is V-shaped (Figure 2). In this paper we refer to the angle of the V-shape as filter angle .The further a point is over the connection line of another point, the more likely it is to be a vegetation point. Therefore the elimination area should have a greater filter angle.

For example: If the filter angle is 80 degrees; a point located one meter above the connecting line of another point would be classified as a non-ground point if a lateral deviation of 17.36 cm is not exceeded. A point only half a meter above the connecting line is classified as such only up to a lateral deviation of 8.68 cm. If a point is within this elimination area, it is marked as a non-ground point and is eliminated from further calculations. If a point is outside this elimination area, it is recognized as a ground point and used as a basis for a new elimination area.

**Figure 3.**
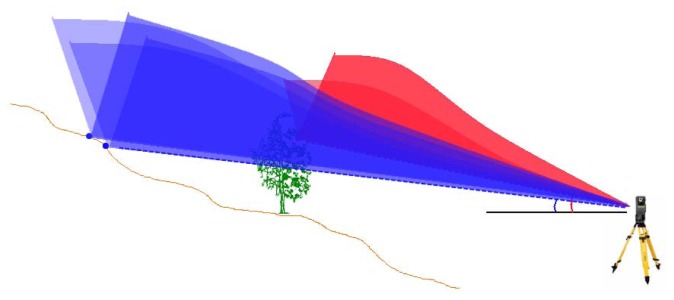
Oblique view of a terrestrial laser scan. The elimination areas are shown three-dimensionally and thus appear in the form of wedges.

The angle of the V-shape is the primary parameter for the filtering procedure. If the inclination of the elimination area is flatter than the terrain inclination, even terrain points are eliminated. For any point an upward open wedge is generated (Figure 3). Any point that falls into the wedge of another point is eliminated as non-ground point. The wedges in Figure 3 we present in a slightly oblique view to emphasize the V-shape. For the elimination areas, also forms other than the V-shape are possible, for example when the horizontal deviation increases exponentially or according to a specific function curve to the difference of the vertical angle.

**Figure 4.**
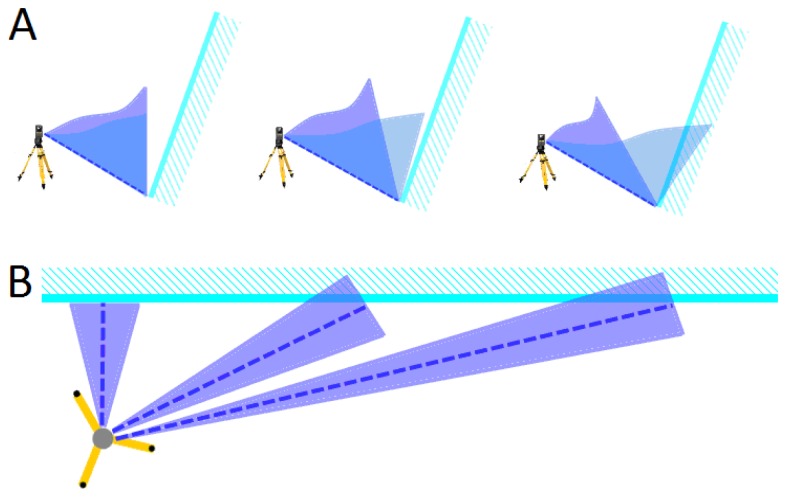
Laser scan of a wall with three different horizontal angles; seen from the side (**A**) and from above (**B**).

It is important to consider the different filtering behaviour for long and steep elements, for example for walls. For visualization, we present three laser scan records shown from above and from the side. Laser beams hit a slightly inclined wall (Figure 4, light blue line) from different horizontal angles.

If the recording beams hit the wall perpendicular (Figure 4(A,B), left), the filter angles have no influence on the wall and only exactly vertical and overhanging parts are eliminated (2.5 D filter). In case of the situation shown in the middle of Figure 4(A) and (B), the filter angle would have a larger influence. In the recording on the right side of Figure 4(A) and (B), where the wall is nearly in the direction of the laser beam, the wall would be filtered out if it was only minimally steeper than the filter angle.

## 3. Description of Test Areas

### 3.1. First Test Area

The test area is located near the village of Gries am Brenner in Tyrol, Austria. In addition to the automated filtering, we manually categorized points into ground and non-ground points. The manually filtered results are therefore suitable for comparison with results from automated filtering methods.

**Figure 5.**
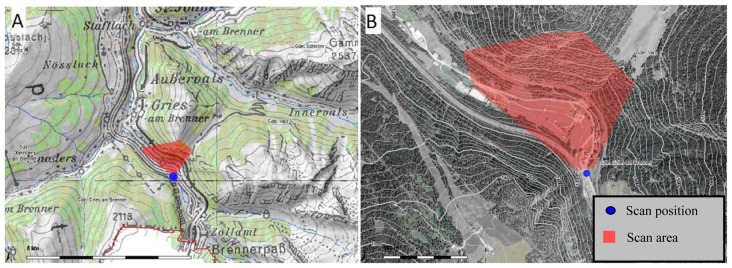
Test are ((**A**): ÖK 50; (**B**) aerial photo); image source: © Land Tirol, *tiris*, www.tirol.gv.at/tiris.

**Figure 6.**
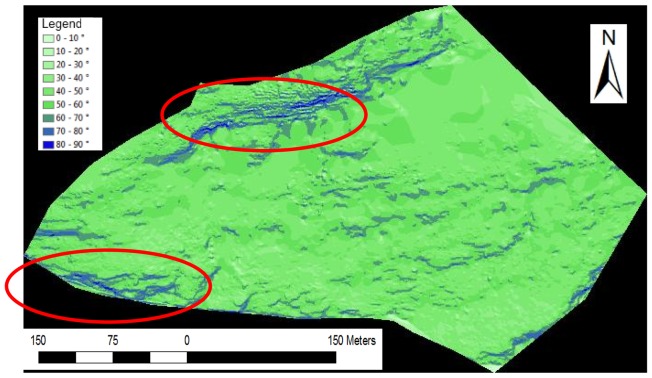
Inclination map of the test area, generated with the “slope” function of ArcGIS.

We undertook scanning from three positions. The surveyed areas only overlap slightly. For testing, we chose a record with 114,800 points. We took measurements of the south-facing slopes of the Padauner Kogel (Figure 5). The terrain in the approximately 1 km long area has a height difference between valley and mountain tops of approximately 450 m and covers an area of 0.3 km^2^. Areas with vegetation alternate with up to 70 degree steep cliffs without vegetation. The terrain is diverse and has abrupt terrain transitions. Here, automated filtering of the laser scanning data is difficult. Most filtering methods work with angle thresholds and these thresholds must be high in order not to falsely eliminate the existing natural terrain transitions. Figure 6 shows an inclination map of the test site. Flat areas are green to account for the prevailing forest cover. The mostly barren slopes with more than 60 degrees inclination remain blue. Two significant, large rock formations are on the upper side and at the left below.

### 3.2. Second Test Site

**Figure 7.**
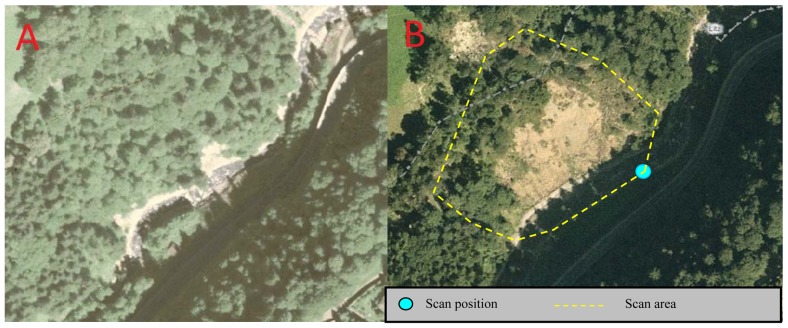
Aerial photos Galierm, 2001 (**A**) und 2006 (**B**); image source: © Land Vorarlberg, http://vogis.cnv.at.

**Figure 8.**
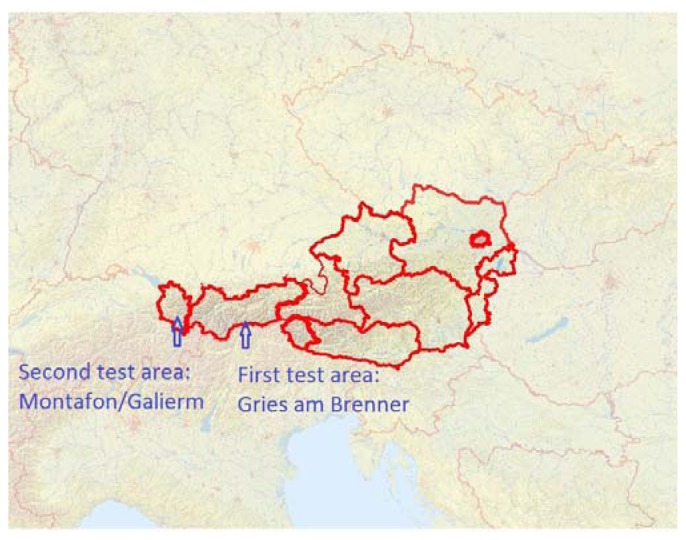
Location of the two test areas; image source: © Geoland, www.geoland.at.

The study area is located in the valley of Montafon near Schruns in Vorarlberg, Austria (Figures 7 and 8). We chose one scan position, at a distance of approximately 100 m to the scanned slope. The scan contains 196,932 points. In contrast to the first test area, the filtering is easier, because of significantly reduced vegetation. Only some fallen trees and tree trunks exist due to a landslide.

## 4. DEM Calculation of the Test Areas Using the Wedge-Filtering Method

**Figure 9.**
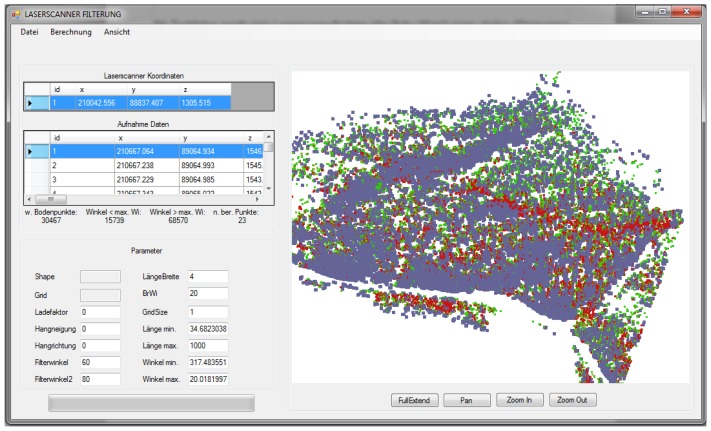
Screenshot of the calculation using our own computer program.

To implement wedge-filtering in a computer program, we used the programming language VB.NET. The filter angle value is the only input parameter for the calculation (Figure 9).

For the evaluation we chose four different filter angles: 80, 70, 60 and 50 degrees. We omitted lower angles, because the slopes in the test areas are generally steeper. We classified every point of the point cloud by the following two steps:

(1) comparison of the horizontal distance of the line between laser scanner and the point to be verified with the distance between scanner to every other point:
  (1) $${\text{distance}}_{\text{pv}}<{\text{distance}}_{\text{pc}}$$
  where: *distance_pv_* is the horizontal distance between the laser scanner and the point to be verified, and *distance_pc_* is the horizontal distance between the laser scanner and the compared point.

If the distance to the point to be verified is longer than the distance to a compared point, the point to be verified cannot be in the wedge of the compared point. Therefore, we cannot determine whether it is a ground point or not. In this case we need to compare the next point of the point cloud. If the distance to the point to be verified is shorter than that to the compared point, the verifying point could be in the wedge of the compared point. In this case, we continue with step 2:

(2) The next step is verifying whether the point is in the wedge or not. We can calculate the angle between the two lines and assess if the angle exceeds the user-defined threshold filter angle:
  (2) $$\text{Atan}(\Delta \theta /[+/-]\Delta \varphi )>\lambda_{\text{thres}}$$
  where: *θ:* azimuth-angles of the points with the origin in the laser scanner; *φ:* polar-angles of the points with the origin in the laser scanner, and *λ_thres_*: threshold-angle which must be defined.

If this angle between the lines to the point to be verified and the compared point is larger than the defined filter angle, the point to be verified is located in the region of the wedge of the compared point and therefore can be classified as a non-ground point. An ASCII file contains the result of the calculation including. X, Y, Z coordinates and a status value for each point. The value “0” indicates a ground point, the value “1” indicates a non-ground point.

To obtain a digital elevation model (DEM), we interpolate the ground points using ArcGIS by ESRI Inc. According to ESRI [19] the Natural Neighbour method is also well suited for distributed point clusters, for example from terrestrial laser scan recordings. Based on the computed results, we calculated four DEMs with a cell size of one meter. To evaluate the results we used DEMs of the test areas, which had been generated with manually filtered ground points. We calculated the difference between the newly calculated and the manually filtered DEMs.

Additionally to the results of the new filter approach we described above, we compared DEMs of the test areas with the results of the automated filter method described by Prokop and Panholzer [14].

To evaluate the accuracy to the reference DEMs, we calculated the mean error and the root-mean-square-error (RMSE). According to the ASPRS Guidelines [1] and Gianinetto and Fassi [20], the RMSE is often used to assess the accuracy of elevation data and is defined as:

(3) $$\text{RMSE}=\sqrt{\frac{\sum_{i=1}^{n}{\left(\Delta Z_{i}\right)}^{2}}{n}}$$

where ΔZ_i_ are the elevation residuals (*i.e*., the differences of the elevation measures with respect to reference data) and n is the number of measures. Höhle *et al.* further recommend to use the median, the normalized median absolute (NMAD), the standard deviation, the 68.3% quantile and the 95% quantile of absolute residuals for accuracy assessment of DEMs [21,22].

## 5. Results and Discussion

### 5.1. First Test Area

The centre of the first test area shows a low point density after we manually filtered the data. Because of the missing ground points, the significance of the filtering effect is low. Consequently, the statistical values for the entire recording area are distorted. To allow for more significant results and to obtain a better comparison, we additionally selected a specific area with a high point density for further analysis.

**Figure 10.**
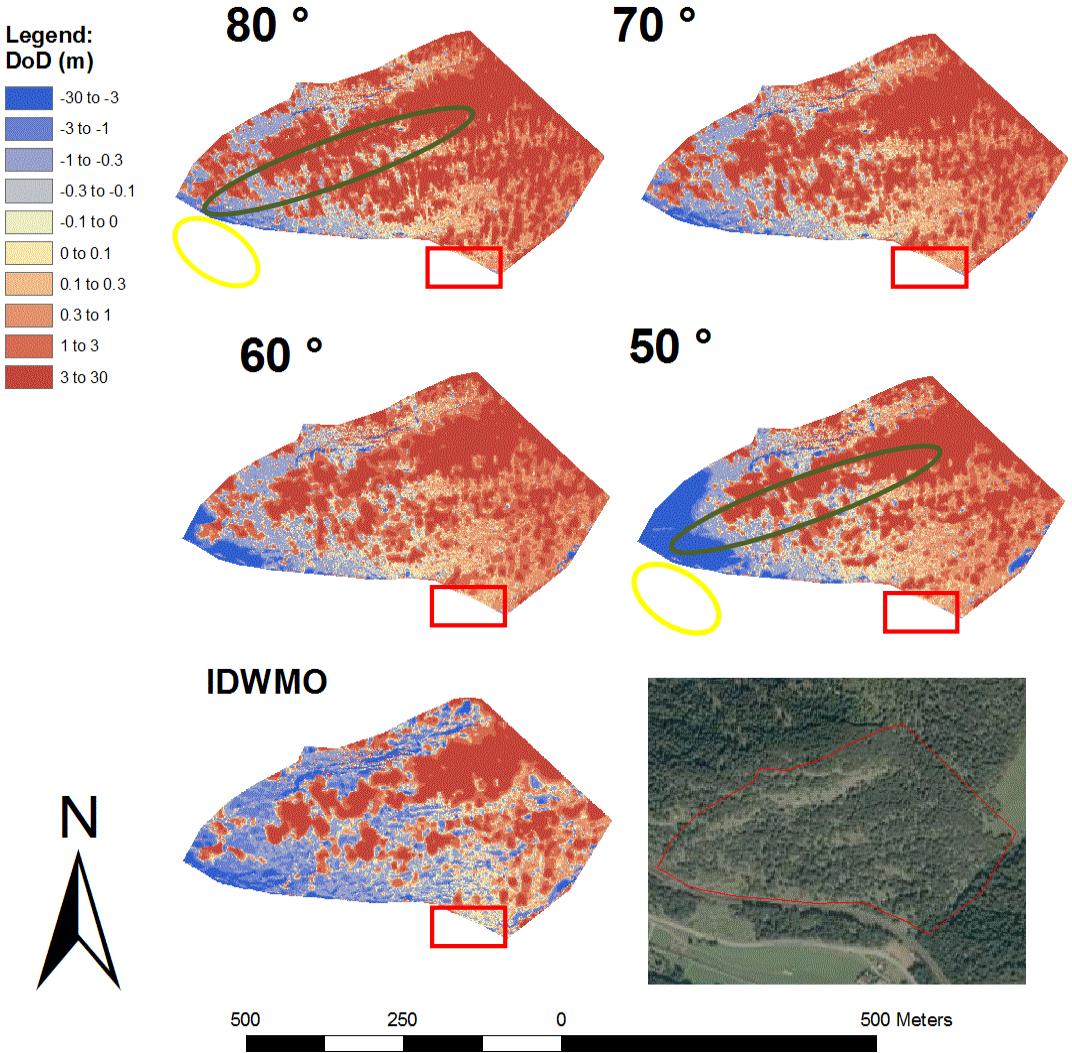
Differences of the first test area between the new calculated terrain models (filter angles 80, 70, 60 and 50 degrees) and the manually filtered terrain model (m). Red rectangle indicates the area of high point density.

In Figure 10 we show the differences between the new calculated DEMs and the manually filtered DEMs (DoD = Differences of DEMs). The areas with warm colour indicate a good accordance between the two DEMs. The red areas indicate where the newly calculated DEMs are located above the reference model, resulting from an insufficient filtering effect. Blue areas show where the newly calculated DEM is lower than the reference model, as a result of incorrectly filtered ground points.

We identified 39,659 ground points and 39,110 non-ground points using a filter angle of 80 degrees. The mean error of the two surfaces is 4.779 m, with a RMSE of 6.996 m and a standard deviation of 5.110 (Table 1). The red areas in the image indicate vegetation, which has not been filtered out. Looking at the pictures in decreasing filter angle order, a reduction in size and number of these red areas is visible. Especially in the forested areas in close proximity to the laser scanner, an area with high point density, a smaller filter angle yields a better filter effect, as indicated by the larger warm coloured zones in Figure 10. In the more distant forested areas the filtering effect is reduced, even when we use a filter angle of 50 degrees (recognizable by the large red areas in the centre of Figure 10). In this area we could only record the highest tops of the trees, since it is unlikely that the laser beam penetrates to the ground of such forested areas. Therefore effective filtering is difficult.

When we use a filter angle of 50 degrees in the test record, the number of ground points is reduced to 24, 398 and the mean error is 1.618 m. This last result must be questioned, because the value of the RMSE is 5.316 m and thus higher than 5.284 m, which is the RMSE at an angle of 60 degrees. The standard deviation even raises from 4.412 m at an angle of 60 degree to 5.064 m at an angle of 50 degree. This decline in accuracy is a result of the steep rock walls at the left side of the recording (Figure 10, yellow lines). When we look at the pictures in decreasing order of the filter angle, the blue areas increase in size. This indicates that ground points were filtered out. This area has slopes partly exceeding 70 degrees. Because the face of the rock wall was measured from the side, the chosen filter angle affects the quality of the filter. Consequently, a slope with 70 degrees inclination - when measured from the side-will be excluded if the filter angle is 60 degrees. In the other rock wall (Figure 10, green lines) there are only small blue stripes visible in the picture, showing the results of a filter angle of 50 degrees. Because the rock wall was recorded frontally, there are only few unwanted filtering effects. The differences between the area calculated with the IDWMO-method and the manually filtered DEM are also shown in Figure 10. The standard deviation is 3.169 m and the RMSE is 3.333 m.

**Figure 11.**
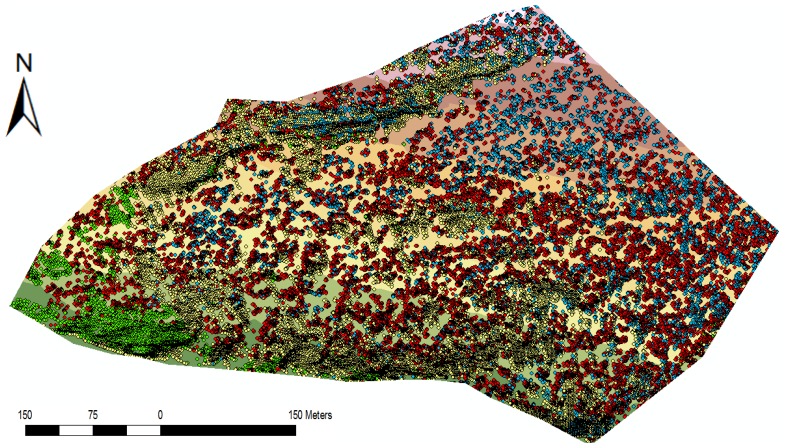
First test area—point classification of the new calculated model (filter angle 50 degrees), compared with the manually filtered terrain model in the form of a colour-separated point cloud.

In Figure 11 colour-separated point clouds compare the results of the point classification of the new model calculated using a filter angle of 50 degrees with the manually filtered terrain. The yellow and blue points were detected as ground points during the wedge-filtering. The 15,867 yellow dots correspond well with the results of the manually filtered terrain model. All 8,561 blue points are located more than 20 cm above the manually filtered terrain model and were thus classified falsely as ground points. The red and green points were classified as non-ground points. The 49,650 red points are all more than 20 cm above the manually filtered terrain model and were thus correctly classified as non-ground points. The 4,721 green points were classified as non-ground points, although they match with the manually filtered terrain model. As mentioned above, a filter angle of 50 degrees would be too low for the left side of the recording. In the area with mainly blue points in the upper third of the picture, however, a lower filter angle would probably yield better results.

Table 2 shows the results of the classification using the filter angles 80, 70, 60 and 50 degree as described above. We can see an increasing filtering effectiveness by decreasing the filter angle from 80 degrees to 50 degrees. The number of undetected non-ground points decreases from 19,988 to 8,561. The number of falsely eliminated ground points increases from 894 at 80 degrees filter angle to 4,721 at 50 degrees filter angle, however.

### 5.2. Detail of First Test Area with a High Point Density

The objective of the following assessment is to understand how the wedge filter works under more homogeneous conditions. Therefore we calculated statistical values also for a sample area of the first test area (Figure 10, red rectangles). The resulting statistical values are summarized in Table 3.

A significant improvement can be seen by reducing the filter angle (see RMSE values in Table 3). The RMSEs of the wedge-filtering method for the entire test area are larger than the RMSEs using the IDWMO method. In the area with higher point density, however, the RMSE of the result using a 50 degree filter angle is 1.180 m the standard deviation 0.962 and therefore lower than RSME and the standard deviation of the IDWMO method with 1.366 m. A higher number of points of the terrain could be preserved using the wedge-filtering approach (see ground points in Table 3).

### 5.3. Second Test Area

**Figure 12.**
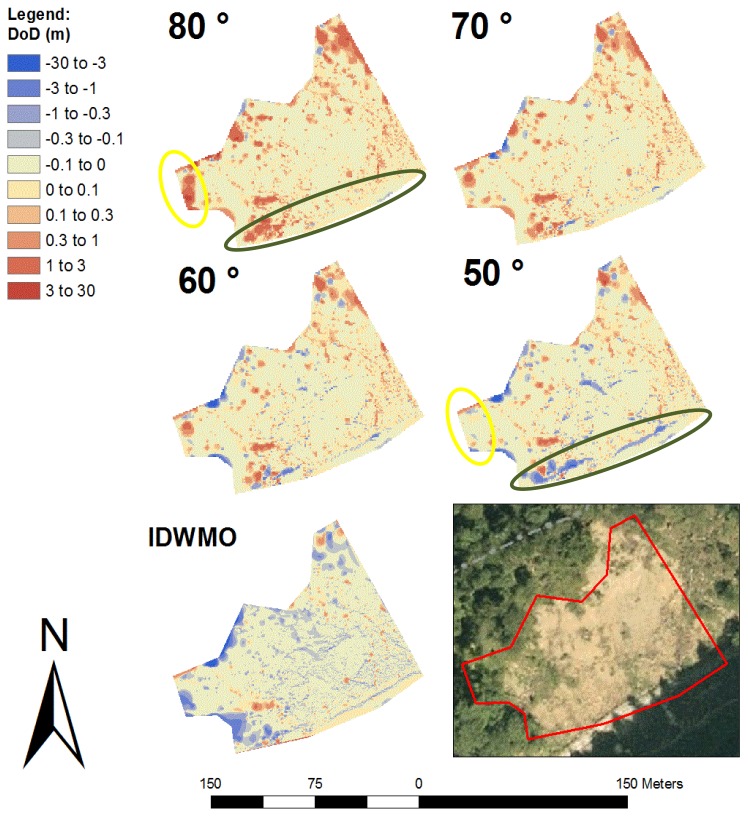
Differences of the second test area between the new calculated terrain models (filter angles 80, 70, 60 and 50 degrees) and the manually filtered terrain model (m).

We calculated, similarly to the first test area, four different DEMs using filter angles of 80, 70, 60 and 50 degrees and determined the difference between the newly calculated elevation models and the manually filtered elevation model (Figure 12 and Table 4). In Figure 12 two regions are highlighted with yellow and green color. The area marked yellow represents densely forested terrain. Applying a filter angle of 80 degrees, not all vegetation points were filtered, shown by the red areas. The filter angle of 50 degree delivered better results, indicated by the warm-coloured areas. The areas marked green represent steep walls. Using a filter angle of 50 degrees, we obtain some black blue, because parts of the wall were filtered out incorrectly (in contrast to the result at 80 degrees).

A filter angle of 70 degrees has proved to show the best results, with an RMSE of 0.387, where over 80,000 of the 196,932 points were filtered out. With the IDWMO method we obtain a RMSE of only 0.517. The higher point density and reduced vegetation result in a much lower average height and RMSE. The 95% quantile of 0.075 m is lower than the 95% quantiles of the wedge filtering. The reason is the morphological opening of the IDWMO method, where a minimum raster is used. As we can see by the low mean error of −0.126 m the IDWMO method tends to filter out more points than wanted. In case of wedge filtering using a filter angle higher than the steepest slope of the scanned area no ground point will by filtered out incorrectly. The high mean errors and 95% quantiles indicate non-ground points which had not been filtered out.

## 6. Conclusions and Outlook

We present a method particularly suited for filtering terrestrial laser scanning data. This method is based on the direct line of sight between the scanner and the measured point and the assumption that no other surface point can be located in the area above this connection line. The analysis of the method shows that even with a high filter angle and associated low error probability we can filter out a considerable number of non-ground points. To filter successfully, the selected filter angle must be as low as possible. The effect of the filter angle differs between measuring an object from the side or frontally. For measurements from the side, the filter angle should not be higher than the maximum inclination of the terrain. A possible approach to improve the quality of the presented filter is dividing a total area into small homogeneous subareas with similar inclination conditions and recording angles.

Our method is a new method for filtering terrestrial laser scanning data, rather than a stand-alone solution for calculating a digital terrain model. The method can be used as a supplement, for pre-filtering, or it can be helpful for the verification of results from other methods. The assumption of the direct line of sight between laser scanner and recorded point is an important additional filter parameter not yet used in other filtering methods. Using wedge-filtering, many points can already with good confidence-be classified as non-ground points. Especially with iterative methods, such as robust interpolation or inverse distance weighting, wedge-filtering can prevent that too many non-ground points are included in the first surface interpolation. Furthermore, wedge-filtering correctly removes some non-ground points, which other methods would incorrectly retain as ground points. For parts of the research area, the mean error as well as the RMSE between the results and the manually filtered reference surface of the compared methods are similar, but a significantly higher number of ground points could be preserved using the wedge-filtering approach.

We only describe the basic method of a new filter approach, however we propose that it is possible to achieve a complete filter based on the function of the wedge-filter, by accounting for additional criteria, even without the use of conventional filter methods. Consequently, in our current work we try to implement iterative calculation methods using thresholds for refractive angles to neighbouring points.

## Figures and Tables

**Table 1.** Statistical values for the first test area.

**Value**	**Filter Angle of Wedge-Filtering**				**IDWMO Method**

	80	70	60	50	
Ground points	39,659	34,108	29,229	24,398	12,326
Mean error	4.779	3.816	2.908	1.618	1.032
RMSE	6.996	5.996	5.284	5.316	3.333
Standard deviation	5.110	4.626	4.412	5.064	3.169
Median	3.304	2.096	1.277	0.747	0.166
NMAD	6.474	5.680	5.085	5.000	3.411
68.3% quantile	7.170	5.360	3.819	2.472	1.258
95% quantile	14.257	12.981	11.765	10.654	7.718

**Table 2.** Point classification of the four new calculated models (80, 70, 60 and 50 degree of filter angle) compared with the manually filtered terrain model.

	**Manual filtering**			

**Filter Angle**	**Ground Points Wedge-Filtering: Ground Points**	**Non-Ground Points Wedge-Filtering: Non-Ground Points**	**Non-Ground Points Wedge-Filtering: Ground Points**	**Ground Points Wedge-Filtering: Non-Ground Points**
80°	19,671	38,216	19,988	894
70°	19,156	43,252	14,952	1,409
60°	17,884	46,862	11,345	2,678
50°	15,837	49,650	8,561	4,721

**Table 3.** Statistical values for the detail area.

**Value**	**Filter Angle of the Wedge-Filtering**				**IDWMO Method**

	80	70	60	50	
Ground points	3,427	3,172	3,014	2945	1,346
Mean error	2.293	1.923	1.296	0.862	0.361
RMSE	4.084	2.819	1.855	1.180	1.366
Standard deviation	3.380	2.353	1.537	0.962	1.366
Median	0.613	0.520	0.456	0.399	-0.054
NMAD	3.705	2.451	1.529	0.911	1.057
68.3% quantile	1.925	1.196	0.828	0.624	0.125
95% quantile	10.123	7.054	4.390	2.618	1.876

**Table 4.** Statistical values for the second test area.

**Value**	**Filter Angle of Wedge-Filtering**				**IDWMO Method**

	80	70	60	50	
Ground points	122,975	116,824	108,186	97,426	120,299
Mean error	0.219	0.092	0.024	−0.030	−0.126
RMSE	0.768	0.387	0.446	0.464	0.517
Standard deviation	0.736	0.376	0.445	0.463	0.502
Median	0.000	0.000	0.000	0.000	−0.058
NMAD	0.499	0.252	0.198	0.225	0.249
68.3% quantile	0.041	0.024	0.008	0.001	−0.035
95% quantile	1.256	0.619	0.412	0.272	0.075
